# Machine learning-driven prediction model for successful weaning of patients from mechanical ventilation in ICU

**DOI:** 10.1186/s40635-026-00859-8

**Published:** 2026-01-21

**Authors:** Changcui Qiu, Lulu Tang, Yugang Zhuang, Chunwei Chi, Kangwei Zheng, Xiaoping Zhu

**Affiliations:** 1 Tongji University School of Medicine, Shanghai, China; 2 Department of Nursing, Shanghai Tenth People’s Hospital, 301# Middle Yanchang Road, Jingan District, Shanghai, 200072 China; 3 Department of Critical Care Medicine, Shanghai Tenth People’s Hospital, Shanghai, China

**Keywords:** Mechanical ventilation, Weaning, Machine learning, Prediction model, Risk factors

## Abstract

**Background:**

Mechanical ventilation is a critical life support technology in the intensive care unit. However, the weaning process remains complex, making the optimal timing for liberation from ventilation challenging to ascertain and imposing a considerable clinical workload. Additionally, advanced weaning assistance tools that integrate multidimensional clinical factors to help clinical staff make precise decisions during the weaning process are lacking. The aim of this study to develop and validate an interpretable machine learning model that comprehensively evaluates the factors influencing weaning to provide clinical decision support for weaning.

**Method:**

We collected data from the ICU of Shanghai Tenth People’s Hospital and its affiliated hospitals. Ten distinct machine learning algorithms for predicting extubation outcomes in patients receiving mechanical ventilation were developed and internally validated. Model performance was quantified using the area under the receiver operating characteristic curve AUC, overall accuracy, sensitivity, specificity, and F1 score. NRI, IDI, and DCA were used to comprehensively identify the optimal model. The relative contribution of each predictor was ranked and compared through SHAP analysis, and the best-performing model was externally validated.

**Results:**

Through univariate and LASSO analyses, 24 predictive variables for machine learning model construction were identified. Comprehensive evaluation showed that among the candidate algorithms, the LGB model demonstrated the highest overall performance. SHAP analysis revealed that the top-ranked features for predicting successful liberation from mechanical ventilation were creatinine levels, lactate levels, the level of consciousness, SpO2, systolic blood pressure, cough reflex, chronic respiratory disease, diastolic blood pressure, and age.

**Conclusions:**

The optimized predictive model, which was developed through the integration of multidimensional predictive factors with diverse machine learning algorithms, exhibits superior predictive accuracy and demonstrates significant clinical potential for determining the optimal timing for weaning patients receiving invasive mechanical ventilation.

*Trial registration*

Current Controlled Trials ChiCTR2400093658; registration date: December 10, 2024.

**Supplementary Information:**

The online version contains supplementary material available at 10.1186/s40635-026-00859-8.

## Introduction

Weaning from mechanical ventilation is a complex and dynamic process that is influenced by multiple factors, including the underlying disease, the patient’s condition, the mode of respiratory support, and the intensity of support [1]. Timely withdrawal from mechanical ventilation is crucial for effective respiratory management and is a key indicator of successful clinical treatment and mechanical ventilation [2, 3].

Research has indicated that approximately 40% of the time spent by healthcare professionals in caring for patients who are on assisted ventilation with invasive ventilators is devoted to preextubation assessment, a process that involves evaluating clinical symptoms, vital signs, respiratory function, and other multidimensional indicators [4]. The assessment process is inherently complex and multifaceted and significantly increases the workload faced by healthcare professionals. Notably, even with adequate preparation, 10–20% of patients who are scheduled for extubation still experience difficulties during or after the procedure or develop dependence on mechanical ventilation. For patients who have been mechanically ventilated for more than 48 h, the extubation failure rate can be as high as 50–70% [5, 6].

In recent years, several studies have reported the development of machine learning (ML)/artificial intelligence (AI)-based models for predicting the outcome of weaning from mechanical ventilation [7–9]. However, machine learning modeling often relies on single sets of retrospectively collected data, and only a few studies have validated their ML models in separate cohorts and assessed the practical benefits of ML in real clinical scenarios. In addition, machine learning methods are singular, there is heterogeneity in the modeling parameters used in machine learning, and many parameters are difficult to obtain in clinical practice [10, 11].

The purpose of this study is to employ multiple machine learning algorithms to construct and validate a mechanical ventilation extubation prediction model for *Broussonetia papyrifera*. After selection of the optimal model, external validation is conducted, and the SHapley Additive exPlanations (SHAP) method is utilized to assess and rank the importance of the model features. Furthermore, the clinical applicability of the optimal model is rigorously evaluated using clinical decision curve analysis (DCA), net reclassification improvement (NRI), and integrated discrimination improvement (IDI) metrics, with the goal of providing a reference for the subsequent promotion and application of the model.

## Methods

### Study population

In this study, data were collected from patients who had been admitted to any of the four intensive care units of Shanghai Tenth People’s Hospital and Chongming Branch between January 1, 2021 and June 30, 2024, these patients served as the modeling group and the internal validation group. An external validation study was concurrently conducted with patients from another intensive care unit at Shanghai Tenth People’s Hospital from December 1, 2024 to July 30, 2025. The inclusion criteria were age ≥ 18 years, hospitalized in the intensive care unit for more than 48 h, and received mechanical ventilation treatment for more than 24 h. The exclusion criteria were having received extracorporeal membrane oxygenation (ECMO) treatment, having advanced cancer, or having families who chose palliative care. All of the patients who were enrolled in this study met the inclusion and exclusion criteria. The study protocol was approved by the Ethics Committee of Shanghai Tenth People’s Hospital and registered with the National Clinical Trial Registration Center (ChiCTR2400093658; registration date: December 10, 2024). This study followed the Transparent Reporting of a Multivariate Prediction Model for Individual Prognosis or Diagnosis (TRIPOD + AI) reporting guidelines [12].

### Sample size calculation

The sample size was calculated using the formula for sample size estimation in predictive modeling described by Riley [13]. In step three, the sample size needed to reduce model overfitting is calculated, assuming an expected shrinkage rate of 10%, with an expected number of variables *P* to be included in the final predictive model of 30: S is the expected shrinkage factor, and the noise ratio evaluation index *R*^2^cs is 0.3; the calculated sample size is 740 cases.

$$\mathrm{n}=\frac{\mathrm{P}}{\left(\mathrm{S}-1\right)\mathrm{ln}\left(1-\frac{{\mathrm{R}}_{\mathrm{CS}}^{2}}{\mathrm{S}}\right)}.$$

This study included 1371 patients in the test group and the internal validation group and 402 patients in the external validation group.

### Primary outcome indicators

The primary outcome was successful weaning or separation (for intubated patients). Successful weaning was defined as extubation without death or reintubation within the next 7 days regardless of whether or not postextubation noninvasive ventilation (NIV) was used or ICU discharge without invasive mechanical ventilation within 7 days, whichever occurred first. The date of successful weaning was counted retrospectively to the actual day of extubation after 7 days without reintubation (or after the patient was discharged if before 7 days without reintubation). For tracheostomized patients, successful weaning or separation was defined as spontaneous ventilation through the tracheostomy without any mechanical ventilation for 7 consecutive days or discharge with spontaneous breathing, whichever occurred first [14].

### Data collection and processing

To identify predictive features and construct a model, we initially constructed a knowledge graph of the factors that influence weaning in patients receiving mechanical ventilation through a literature review. On this basis, in combination with clinical practice, we collected clinical and laboratory data and data on medication use. The data included demographic characteristics, comorbidities, vital signs, treatment measures, blood gas parameters, the results of routine blood tests, biochemical indicators, and coagulation parameters. All the data were obtained through the hospital’s electronic information system. The demographic characteristics and comorbidities of the patients were extracted from structured fields, with comorbidities defined as those mentioned in the patient’s prior medical history and existing diagnoses prior to extubation. The data on vital signs were the worst values recorded within 24 h prior to extubation. Treatment interventions during the period of invasive mechanical ventilation were documented. Blood gas parameters, complete blood count, biochemical parameters, and coagulation profiles were obtained from the most recent measurements taken immediately before extubation in patients with oral intubation or prior to the discontinuation of ventilatory support in patients with tracheostomy. Variables with a missing data rate exceeding 25% were excluded to reduce bias, and the remaining variables were imputed using the multiple imputation by chained equations (MICE) method [15].

### Model development and validation

The current models were developed using ten classifiers, including K-nearest neighbors (KNN), support vector machine (SVM), neural network (NN), extreme gradient boosting (XGB), logistic regression (LR), random forest (RF), decision tree (DT), extra trees (ET), stochastic gradient descent (SGD), and light gradient boosting (LGB). ML models were built by training datasets with target attributes and preprocessing features. Before training, the dataset was randomly divided into a training set and a test set at a ratio of 7:3 and then standardized [16]. Each model’s performance was evaluated and compared using metrics such as accuracy, precision, recall, F1 score, and the area under the curve (AUC). The best-performing model was selected as the final predictor on the basis of the evaluation results [17]. In the classification confusion matrix definition, successful weaning and unsuccessful weaning were considered true positive (TP) and true negative (TN), respectively, if they were accurately predicted by the machine learning model; if they were not correctly predicted by the model, they were considered false positive (FP) or false negative (FN) [18]. The specific definitions of these parameters are as follows: accuracy = (TP + TN)/(TP + TN + FP + FN). Sensitivity = TP/ (TP + FN). Specificity = TN / (TN + FP). F1 score = 2 * Precision * Recall / (Precision + Recall).

### Statistical analysis

All the statistical analyses were performed using R version 4.4.2 and Python version 3.8. Continuous variables are presented as the means [standard deviation (SD)] or medians [25th, 75th percentiles], and categorical variables are presented as counts (percentages) (%). To simplify the model building process, t tests, Mann‒Whitney U tests, and chi‒square tests were used to identify variables that showed statistically significant differences between the success and failure exit groups. Additionally, dimensionality reduction was performed using the least absolute shrinkage and selection operator (LASSO) combined with fivefold cross-validation to identify the optimal predictor variables for the machine learning models. The best variables were incorporated into the machine learning models, and a systematic analysis of ten classifiers was conducted. After comprehensive evaluation of the classifiers, the model with the best predictive performance was selected. The imbalance between the external validation dataset and the internal validation/training sets was mitigated using the SMOTE algorithm. The area under the curve (AUC) of the predictive model was obtained by plotting the ROC curve, and the calibration curve was used for further evaluation of the model’s predictive ability. The SHAP method was employed to analyze feature importance, and the clinical applicability of the model was assessed using DCA, INR, and IDI values. In two-tailed tests, a* p*-value < 0.05 was considered to indicate statistical significance.

## Results

### Patient characteristics

**Fig. 1:**
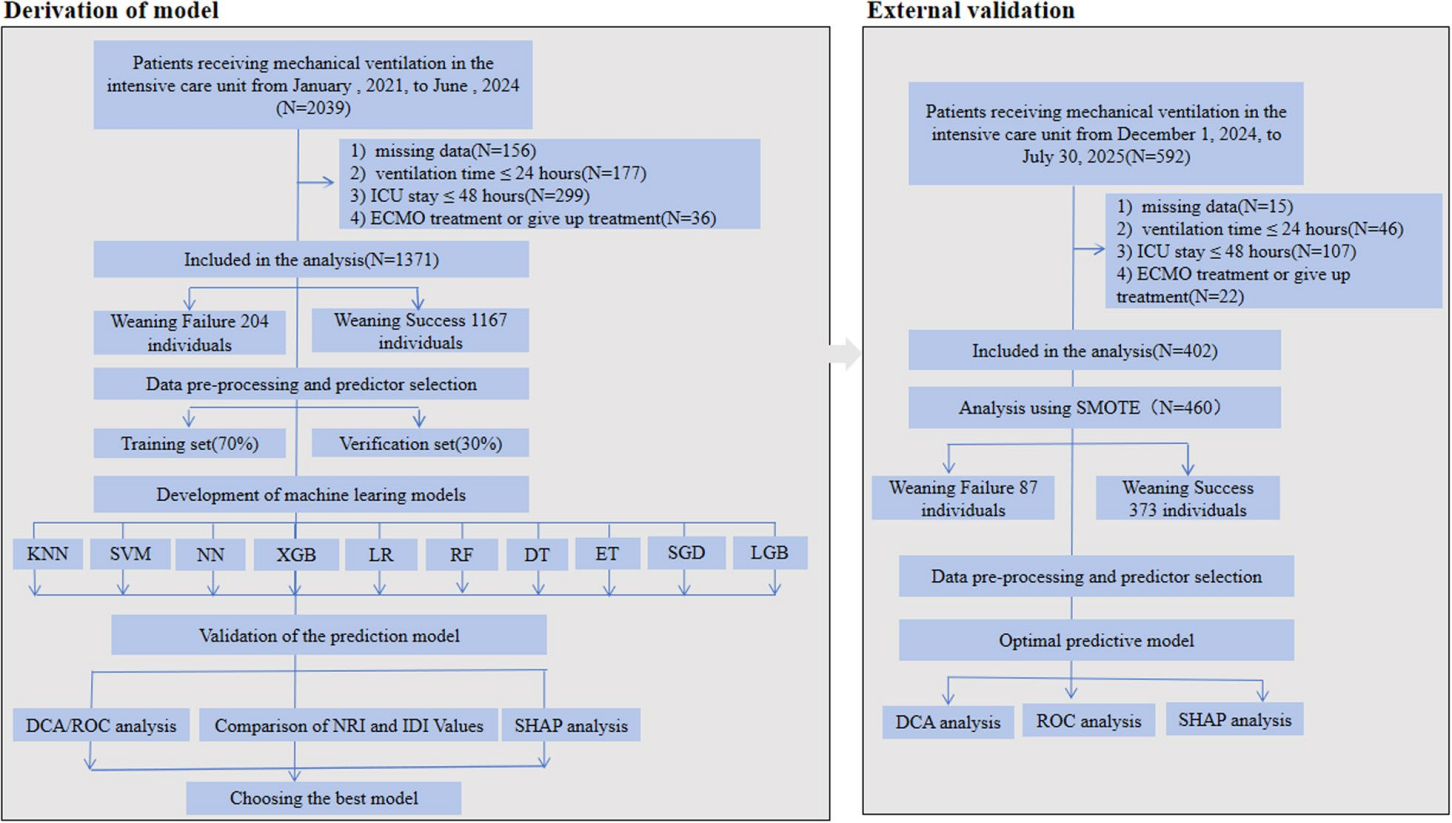
Research flowchart. This flowchart illustrates the patient screening and machine learning model development process. Ten classifiers were used to develop the machine learning models. The models were trained and internally validated at a 7:3 ratio, and the optimal model was selected for external validation following SMOTE analysis

The training and internal validation sets in this study initially included 2039 patients who received mechanical ventilation; 512 of these patients did not meet the inclusion and exclusion criteria, and approximately 156 patients for whom more than 25% of the data were missing were excluded (Fig. 1). Table 1 shows a comparison of 47 demographic and clinical characteristics in patients with different device withdrawal outcomes. Of the 1371 participants, 1167 (85.12%) were successfully weaned from the ventilator. The average age of the subjects was 66.33 years, and 68.9% were male. Compared with patients in the successful weaning group, patients in the weaning failure group were older, and had longer ICU stays, and longer durations of mechanical ventilation. Preliminary analysis revealed significant differences between the two groups in the following factors (*P* < 0.05): ① six sociodemographic factors: age, duration of ICU stay, duration of mechanical ventilation, surgical history, history of heart disease, and history of chronic respiratory diseases; ② five comorbidity factors: hyperglycemia, hypertension, hemodynamic instability, pulmonary hypertension, and kidney disease; ③ eight vital sign factors: level of consciousness, heart rate, systolic blood pressure, diastolic blood pressure, body temperature, blood oxygen saturation, cough reflex, and sputum viscosity; ④ three treatment measures: early rehabilitation, use of positive inotropes and/or vasoactive drugs, and methods of nutritional support; and ⑤ twelve laboratory indicators (blood gas and biochemical indicators): potential of hydrogen(pH), partial pressure of oxygen(PO₂), and calcium(Ca), lactate, glucose, hemoglobin, potassium(K), albumin, creatinine, cardiac troponin T(cTnT), N-terminal Pro B-type natriuretic peptide (NT-proBN), and Procalcitonin(PCT) levels. Table S1 presents a comparison of demographic and clinical characteristics of the patients in the training set and those in the internal validation set. A total of 960 subjects were included as the training set, with 411 as the internal validation set, at a ratio of 7:3. The current results indicate that there are no statistically significant differences between the training and validation sets in the predictive variables (all *P* > 0.05). The external validation set initially included 592 patients who were receiving mechanical ventilation; of these 190 were excluded, leaving 402 patients (Fig. 1). The demographic and clinical characteristics of all participants in the external validation set are shown in Table S2.

**Table 1:** Demographic and clinical characteristics of successful and failed weaning groups

Variable	Total	Weaning failure	Weaning success	*P*
*N*	1371	204	1167	
General				
Age	66.33 (59–76)	72 (64–80.25)	68 (58–74)	< 0.001
Gender				
Male, *n* (%)	944 (68.85%)	152 (74.51%)	792 (67.87%)	0.071
Female, *n* (%)	427 (31.15%)	52 (25.49%)	375 (32.13%)	
BMI, *n* (%)				
< 18.5	190 (13.9%)	36 (17.65%)	154 (13.2%)	0.191
18.5 ≤ BMI < 24.9	851 (62.1%)	127 (62.25%)	724 (62.04%)	
25 ≤ BMI < 29.9	238 (17.4%)	32 (15.69%)	206 (17.65%)	
≥ 30	92 (6.7%)	9 (4.41%)	83 (7.11%)	
Marriage status, *n* (%)				
Married	1220 (89%)	188 (92.16%)	1032 (88.43%)	0.148
Other	151 (11%)	16 (7.84%)	135 (11.57%)	
ICU time, *n* (%)				
2–7 d	541 (39.5%)	63 (30.88%)	478 (40.96%)	< 0.001
7–28 d	730 (53.2%)	112 (54.9%)	618 (52.96%)	
> 28 d	100 (7.3%)	29 (14.22%)	71 (6.08%)	
Ventilation time, *n* (%)				
1–3 d	503 (36.7%)	48 (23.53%)	455 (38.99%)	< 0.001
3–7 d	286 (20.9%)	34 (16.67%)	252 (21.59%)	
≥ 7 d	582 (42.5%)	122 (59.8%)	460 (39.42%)	
Surgical history, *n* (%)				
No	960 (70%)	116 (56.86%)	844 (72.32%)	< 0.001
Yes	411 (30%)	88 (43.14%)	323 (27.68%)	
Alcohol consumption history, *n* (%)				
No	1289 (94%)	191 (93.63%)	1098 (94.09%)	0.924
Yes	82 (6%)	13 (6.37%)	69 (5.91%)	
Smoking history, *n* (%)				
No	1242 (90.6%)	179 (87.75%)	1063 (91.09%)	0.168
Yes	129 (9.4%)	25 (12.25%)	104 (8.91%)	
Heart disease, *n* (%)				
No	803 (58.6%)	74 (36.27%)	729 (62.47%)	< 0.001
Yes	568 (41.4%)	130 (63.73%)	438 (37.53%)	
HCRD, *n* (%)				
No	934 (68.1%)	85 (41.67%)	849 (72.75%)	< 0.001
Yes	437 (31.9%)	119 (58.33%)	318 (27.25%)	
Comorbidities				
Diabetes, *n* (%)				
No	1059 (77.2%)	132 (64.71%)	927 (79.43%)	< 0.001
Yes	312 (22.8%)	72 (35.29%)	240 (20.57%)	
Hypertension, *n* (%)				
No	773 (56.4%)	93 (45.59%)	680 (58.27%)	0.001
Yes	598 (43.6%)	111 (54.41%)	487 (41.73%)	
Hemodynamic instability, *n* (%)				
No	1164 (84.9%)	125 (61.27%)	1039 (89.03%)	< 0.001
Yes	207 (15.1%)	79 (38.73%)	128 (10.97%)	
Pulmonary arterial hypertension, *n* (%)				
No	1046 (76.3%)	168 (82.35%)	878 (75.24%)	0.034
Yes	325 (23.7%)	36 (17.65%)	289 (24.76%)	
Comorbid neurological disorders, *n* (%)				
No	1027 (74.9%)	146 (71.57%)	881 (75.49%)	0.269
Yes	344 (25.1%)	58 (28.43%)	286 (24.51%)	
Kidney disease, *n* (%)				
No	1248 (91%)	169 (82.84%)	1079 (92.46%)	< 0.001
Yes	123 (9%)	35 (17.16%)	88 (7.54%)	
Vital Signs				
Muscle strength (grade), *n* (%)				
0	17 (1.2%)	3 (1.47%)	14 (1.2%)	0.169
I & II & III & IV	145 (10.6%)	14 (6.86%)	131 (11.23%)	
V	1209 (88.2%)	187 (91.67%)	1022 (87.57%)	
State of consciousness, *n* (%)				
Alert	915 (66.7%)	61 (29.9%)	854 (73.18%)	< 0.001
Drowsy & Stupor & Coma	331 (24.1%)	110 (53.92%)	221 (18.94%)	
Agitated & Sedated	125 (9.1%)	33 (16.18%)	92 (7.88%)	
Heart rate, beats per minute	89 (77–101)	85 (70–103.25)	89 (78–101)	0.006
Systolic pressure, mmHg	128 (114–143)	122 (102–130)	130 (115–145)	< 0.001
Diastolic pressure, mmHg	68.43 (59–75)	64 (55–70)	67 (59–77)	< 0.001
Body temperature, °C	36.9 (36.5–37.2)	36.6 (36.4–37)	36.9 (36.5–37.2)	< 0.001
RSBI	38.52 (32–42)	36 (31.5–42)	36 (32–42)	0.744
Blood oxygen saturation	98.1 (98–99.6)	98.3 (95.2–99.3)	99.1 (98.3–99.6)	< 0.001
Cough reflex, *n* (%)				
0	149 (10.9%)	91 (44.61%)	58 (4.97%)	< 0.001
1	61 (4.4%)	2 (0.98%)	59 (5.06%)	
2	316 (23%)	48 (23.53%)	268 (22.96%)	
3	845 (61.6%)	63 (30.88%)	782 (67.01%)	
Sputum viscosity, *n* (%)				
I	728 (53.1%)	89 (43.63%)	639 (54.76%)	0.013
II	594 (43.3%)	107 (52.45%)	487 (41.73%)	
III	49 (3.6%)	8 (3.92%)	41 (3.51%)	
Therapeutic measures				
Early rehabilitation (during the intubation period), *n* (%)				
No	1303 (95%)	186 (91.18%)	1117 (95.72%)	0.010
Yes	68 (5%)	18 (8.82%)	50 (4.28%)	
Systemic steroid treatment, *n* (%)				
No	1354 (98.8%)	200 (98.04%)	1154 (98.89%)	0.506
Yes	17 (1.2%)	4 (1.96%)	13 (1.11%)	
Use of positive inotropic and vasoactive drugs, *n* (%)				
No	865 (63.1%)	79 (38.73%)	786 (67.35%)	< 0.001
Yes	506 (36.9%)	125 (61.27%)	381 (32.65%)	
Use of sedatives, *n* (%)				
No	795 (58%)	108 (52.94%)	687 (58.87%)	0.132
Yes	576 (42%)	96 (47.06%)	480 (41.13%)	
Nutritional support method, *n* (%)				
Enteral	819 (59.7%)	102 (50%)	717 (61.44%)	< 0.001
Enteral and parenteral	317 (23.1%)	87 (42.65%)	230 (19.71%)	
Parenteral	143 (10.4%)	7 (3.43%)	136 (11.65%)	
Nothing by mouth	92 (6.7%)	8 (3.92%)	84 (7.2%)	
Laboratory Indicators				
pH	7.41 (7.38–7.45)	7.38 (7.29–7.44)	7.43 (7.39–7.46)	< 0.001
PaO_2_, mmHg	131 (100–155.2)	102.5 (81–143.25)	128.3 (100.7–156.8)	< 0.001
Calcium, mmol/L	1.17 (1.12–1.21)	1.13 (1.08–1.21)	1.17 (1.12–1.21)	< 0.001
PaCO₂, mmHg	41.27 (36.4–44.6)	39.15 (34.33–46.4)	40 (36.7–44.2)	0.145
Lactate, mmHg	2.35 (1.2–2.4)	2.9 (1.575–5.325)	1.7 (1.2–2.3)	< 0.001
Glucose, mmol/L	10.04 (7–11.6)	8.2 (6.43–11.33)	9.3 (7.15–11.6)	0.005
Hemoglobin, g/dL	91.24 (73–111)	78 (58–104)	91 (74–112)	< 0.001
Potassium, mmol/L	4.02 (3.66–4.29)	4.1 (3.8–4.71)	3.93 (3.66–4.22)	< 0.001
Sodium(Na), mmol/L	141.54 (138–145)	140 (136.38–15)	141 (138–145)	0.718
White blood cells,*10^9/L	14.75 (7.44–13.31)	10.15 (6–15.4)	9.84 (7.51–12.96)	0.917
Albumin, g/L	33.43 (30.2–36.1)	31.2 (27.2–35.5)	33.5 (30.7–36.25)	< 0.001
Creatinine, μmol/L	107.90 (55.2–108.6)	115.6 (77.5–246.5)	69.4 (53.6–99.6)	< 0.001
Troponin T, μg/L, *n*%				
< 0.1	1016 (74.1%)	103 (50.49%)	913 (78.2%)	< 0.001
0.1–1.0	276 (20.1%)	76 (37.25%)	200 (17.14%)	
> 1.0	79 (5.8%)	25 (12.25%)	54 (4.63%)	
NT-proBN, ng/L, *n* (%)				
< 125	368 (26.8%)	31 (15.2%)	337 (28.88%)	< 0.001
125–450	235 (17.1%)	19 (9.31%)	216 (18.51%)	
450–900	172 (12.5%)	17 (8.33%)	155 (13.28%)	
> 900	596 (43.5%)	137 (67.16%)	459 (39.33%)	
Procalcitonin, ng/L, *n* (%)				
< 0.1	173 (12.6%)	9 (4.41%)	164 (14.05%)	< 0.001
0.1–0.5	698 (50.9%)	84 (41.18%)	614 (52.61%)	
0.5–2	252 (18.4%)	29 (14.22%)	223 (19.11%)	
> 2	248 (18.1%)	82 (40.2%)	166 (14.22%)	

Cough reflex grading: 0 = No cough reflex, even with strong stimulation; 1 = cough reflex in response to airway stimulation but no sputum in the artificial airway; 2 = cough reflex in response to airway stimulation, sputum only in the artificial airway, and the need for suction assistance to help the patient clear it; 3 = no stimulation needed; sputum can be expelled by the patient’s own effort; *RSBI* Rapid Shallow Breathing Index, *HCRD* history of chronic respiratory diseases. The data are presented as the mean (SD), the median [IQR], or n (%)

### Screening of modeling variables based on LASSO regression analysis

Here, we used LASSO regression to screen and reduce the dimensions of the 34 variables with statistically significant differences, as shown in Table 1 [19]. The results show that as the parameter logλ increased, the regression coefficients continuously converged, eventually reaching zero. When the λ of the minimum standard error was 0.05, the continuous variables selected for the Gaussian model were age, systolic pressure, diastolic pressure, PH, PaO2, and Ca, lactate, K, and creatinine levels. When the λ of the minimum standard error was 0.038, the categorical variables for the binomial model were Matrimony2, ICUtime3, ventilationtime3, surgical history, heart disease, history of chronic respiratory diseases, hemodynamic instability, consciousness state2, cough reflex1, cough reflex2, cough reflex3, nutritional support method2, troponin T2, NT-proBN4, and procalcitonin4. Finally, we selected 24 predictor variables and used those variables to develop the machine learning model (Fig. S1 and Table S3).

### Development of a predictive machine learning model

**Fig. 2:**
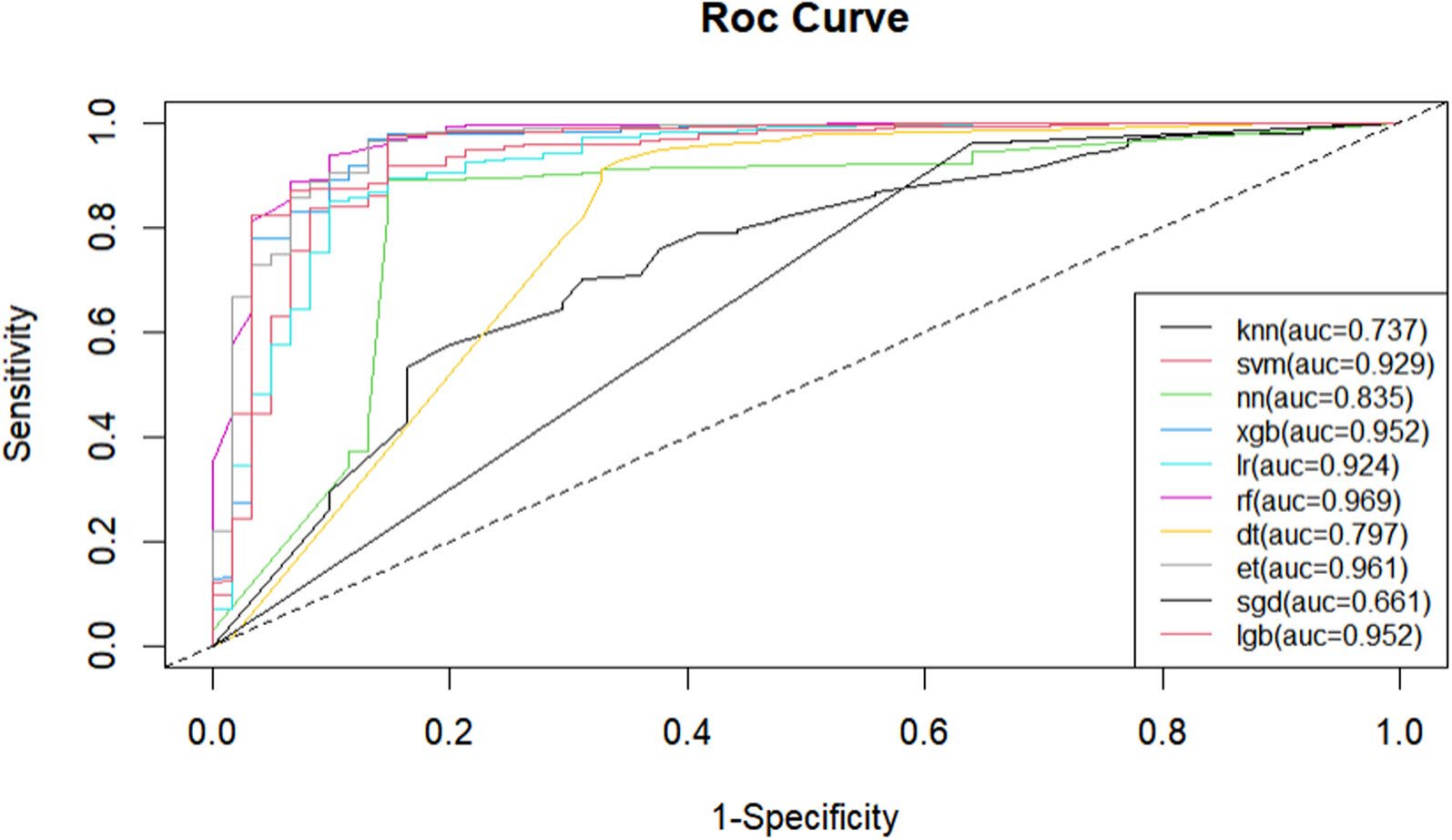
Comparison of the ROC curves for the 10 machine learning models. *AUC* area under the curve, *knn* K-Nearest Neighbors, *svm* Support Vector Machine, *nn* Neural Network, *xgb* Extreme Gradient Boosting, *lr* Logistic Regression, *rf* Random Forest, *dt* Decision Tree, *et* Extra Trees, *sgd* Stochastic Gradient Descent, *lgb* Light Gradient Boosting Machine

The performance of the prediction model is shown in Table 2. The results of the current analysis indicate that the best models are the LGB model and the RF model. The LGB model has an AUC value of 0.952, an accuracy of 0.954, a sensitivity of 0.980, a specificity of 0.803, an F1 score of 0.973, and a precision of 0.966. The RF model has an AUC value of 0.968, an accuracy of 0.956, a sensitivity of 0.983, a specificity of 0.803, an F1 score of 0.975, and a precision of 0.966. ROC curves (Fig. 2) were used to evaluate the value of the model in predicting weaning. The probability of successful weaning predicted by the model was positively correlated with the actual probability of success, and the model demonstrated good calibration (*P* < 0.05). Given that prediction of successful weaning from mechanical ventilation in clinical patients involves a large amount of data, the LGB model was chosen as the optimal model in this study.

**Table 2:** Performance of the developed models based on ten classifiers

Classifier/Performance	AUC	Accuracy	Sensitivity	Specificity	F1 score	Precision
KNN	0.737	0.844	0.977	0.082	0.914	0.859
SVM	0.929	0.915	0.971	0.590	0.951	0.932
NN	0.835	0.886	0.891	0.852	0.930	0.972
XGB	0.952	0.949	0.977	0.787	0.970	0.963
LR	0.924	0.929	0.971	0.689	0.959	0.947
RF*	0.968	0.956	0.983	0.803	0.975	0.966
DT	0.797	0.900	0.965	0.525	0.943	0.921
ET	0.961	0.939	0.989	0.656	0.965	0.943
SGD	0.661	0.873	0.963	0.361	0.928	0.896
LGB*	0.952	0.954	0.980	0.803	0.973	0.966

* indicates the best models

*AUC* area under the curve, *KNN* K-Nearest Neighbors, *SVM* Support Vector Machine, *NN* Neural Network, *XGB* Extreme Gradient Boosting, *LR* Logistic Regression, *RF* Random Forest, *DT* Decision Tree, *ET* Extra Trees, *SGD* Stochastic Gradient Descent, *LGB* Light Gradient Boosting Machine

### Comparison of NRI and IDI values

In addition to the ROC curve, we used the NRI and the IDI as the primary comparison metrics to evaluate the reclassification ability and the overall predictive performance of the optimal model. We compared the performance of the LGB model in predicting the probability of successful weaning with that of the other nine models. Our analysis revealed that, with the exception of the RF model, the LGB model was more effective than any of the other models in identifying high-risk patients and that it performed well in overall risk assessment (Table 3).

**Table 3:** Comparison of NRI and IDI between LGB and other models

Model	NRI (Categorical) (95% CI)	*P*	IDI (95% CI)	*P*
LGB	Reference			
KNN	− 0.6115 [− 0.7337– − 0.4894]	< 0.001	− 0.4735[− 0.5281– − 0.4188]	< 0.001
SVM	− 0.0471 [− 0.2291–0.1349]	0.612	− 0.0921 [− 0.1713– − 0.0129]	0.023
NN	− 0.6115 [− 0.7337– − 0.4894]	< 0.001	− 0.169 [− 0.2278– − 0.1101]	< 0.001
XGB	0.5431 [− 0.4178–0.6684]	< 0.001	0.1125 [0.0842–0.1408]	< 0.001
LR	− 0.0955 [− 0.2757 –0.0847]	0.299	− 0.0978 [− 0.1713– − 0.0242]	0.009
RF	− 0.0107[− 0.1356 – − 0.1142]	0.867	− 0.0086 [− 0.0308– − 0.0136]	0.448
DT	− 0.0143 [− 0.1722 –0.1436]	< 0.001	− 0.1665 [− 0.2447– − 0.0883]	3e-05
ET	− 0.3328 [− 0.4832 – − 0.1825]	< 0.001	− 0.1189 [− 0.1685– − 0.0694]	< 0.001
SGD	− 0.1519 [− 0.3616–0.0578]	< 0.001	− 0.2888 [− 0.4268– − 0.1507]	4e-05

*LGB* Light Gradient Boosting Machine, *KNN* K-Nearest Neighbors, *SVM* Support Vector Machine, *NN* Neural Network, *GB* Extreme Gradient Boosting, *LR* Logistic Regression, *RF* Random Forest, *DT* Decision Tree, *ET* Extra Trees, *SGD* Stochastic Gradient Descent

### DCA modeling analysis

**Fig. 3:**
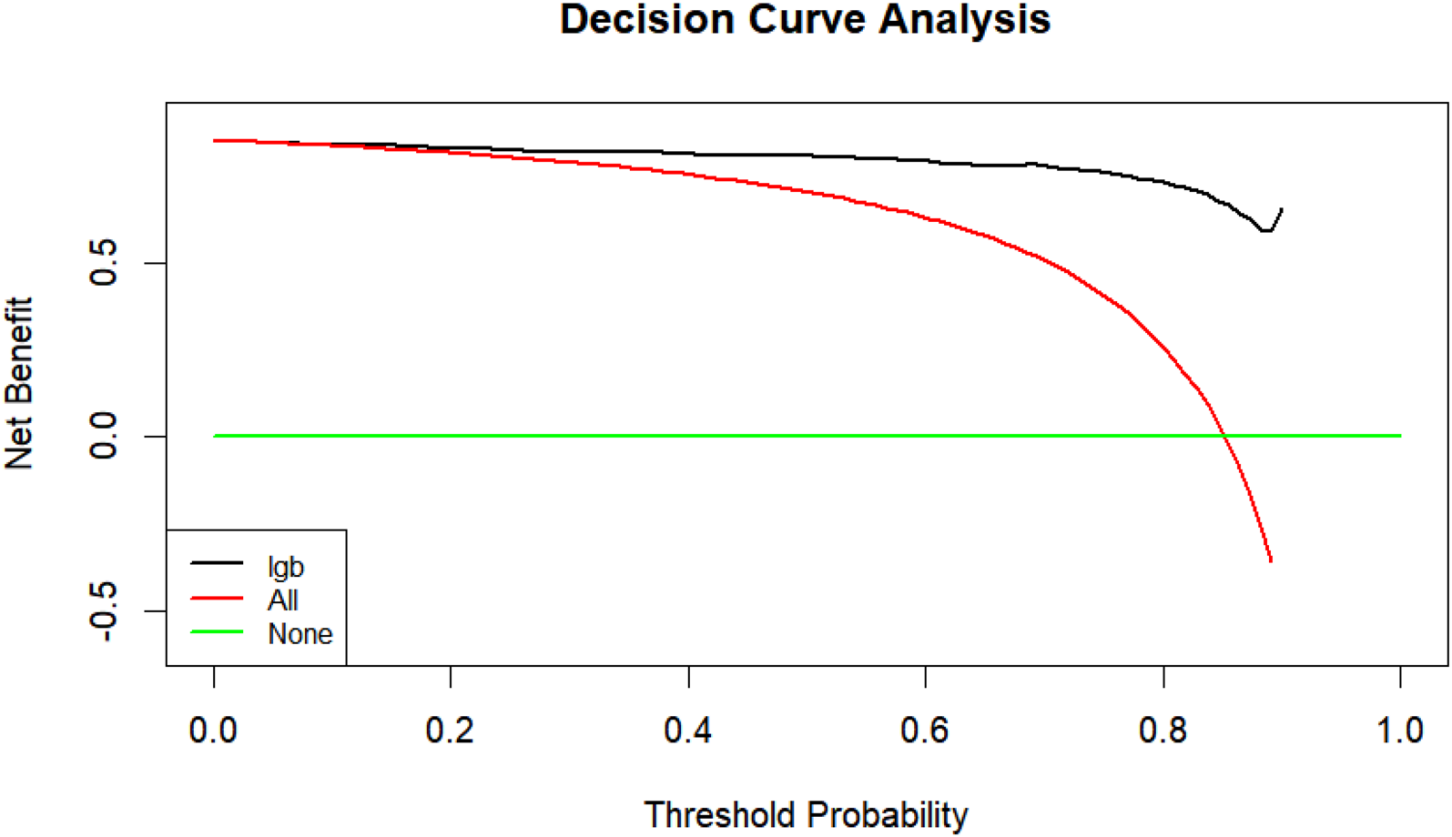
DCA analysis based on the LGB model, *lgb* Light Gradient Boosting Machine

Figure. 3 shows the results of the DCA analysis based on the LGB model. DCA indicated that the LGB model had significant net benefits for threshold probabilities at different time points, suggesting the model’s potential clinical benefit.

### SHAP analysis of the final model

**Fig. 4:**
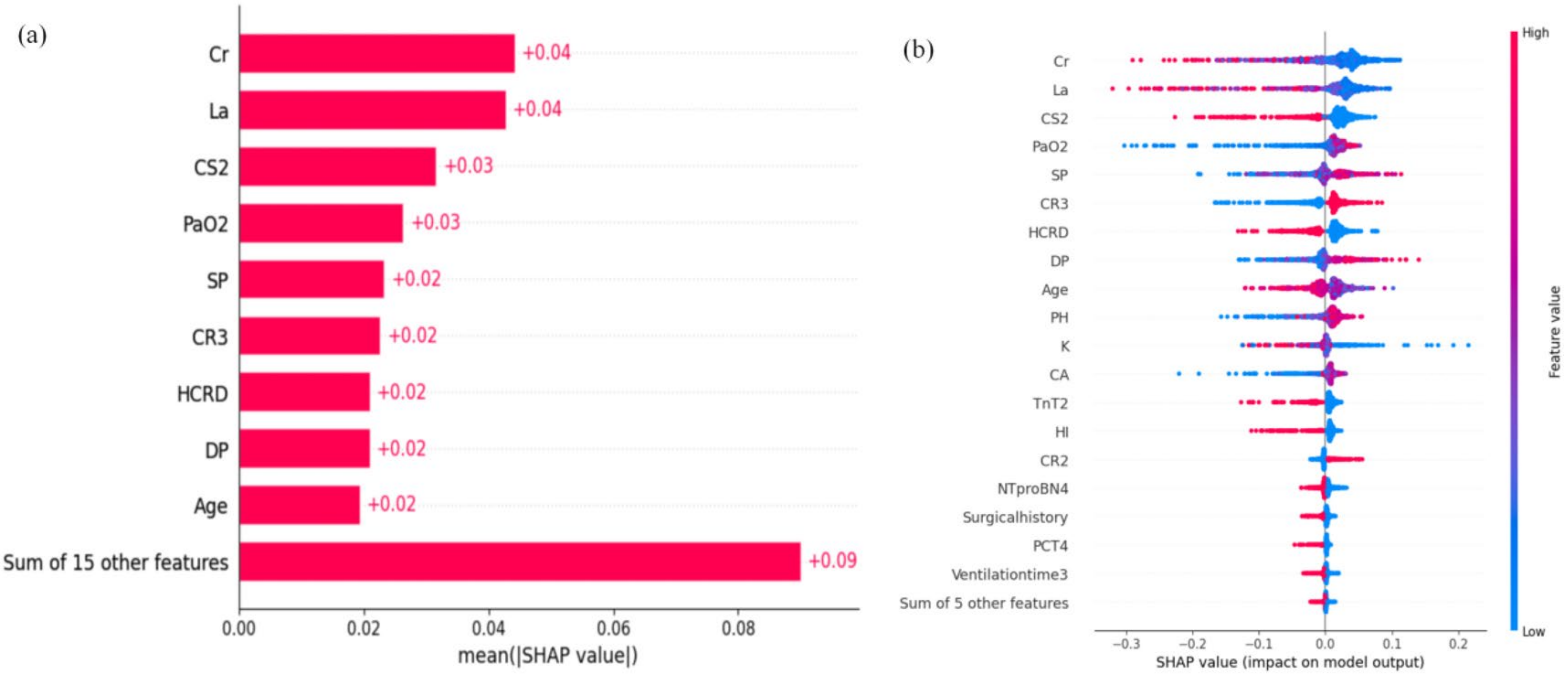
SHAP analysis of the LGB model. **a** SHAP summary bar plot showing feature importance ranking (internal validation); **b** feature importance based on the SHAP results. The features are arranged along the vertical axis, and the SHAP values are plotted on the horizontal axis. The colors of the points for each feature value are different; pink indicates a positive correlation with successful withdrawal, whereas blue indicates a negative correlation with successful withdrawal (internal validation); *Cr* creatinine, *La* lactate, *CS2* consciousness state (Drowsy & Stupor & Coma), *SP* systolic pressure, *CR2* cough reflex (cough reflex in response to airway stimulation, sputum only in the artificial airway and the need for suction to help the patient clear it), *CR3* cough reflex (no stimulation needed to produce a cough reflex; sputum can be expelled by the patient’s efforts alone), *HCRD* history of chronic respiratory disease, *DP* diastolic pressure, *K* potassium, *Ca* calcium, *TnT2* cardiac troponin T (0.1–1.0 μg/L), *HI* hemodynamic instability, *NTproBN4* N-terminal pro B-type natriuretic peptide (> 900 ng/L), *PCT4* procalcitonin (> 2 ng/L)

The importance of all the individual features based on the LGB model and the comprehensive Shapley values are shown in Fig. 4. Each point in the figure represents a feature and its corresponding Shapley value; thus, the figure visually displays the contribution of each feature to the output of the prediction model. The feature values are color coded, and they are arranged on the Y-axis from top to bottom in order of importance. The results of the SHAP analysis indicate that, among patients receiving invasive mechanical ventilation, creatinine is the most important feature for predicting weaning from mechanical ventilation. It is negatively correlated with the Shapley value, indicating that lower creatinine levels make it easier for patients to be weaned off the ventilator. The other important features are lactate levels, the consciousness level, SpO2, systolic blood pressure, cough reflex, chronic respiratory disease, diastolic blood pressure, and age, in that order.

### External validation of the final model

**Fig. 5:**
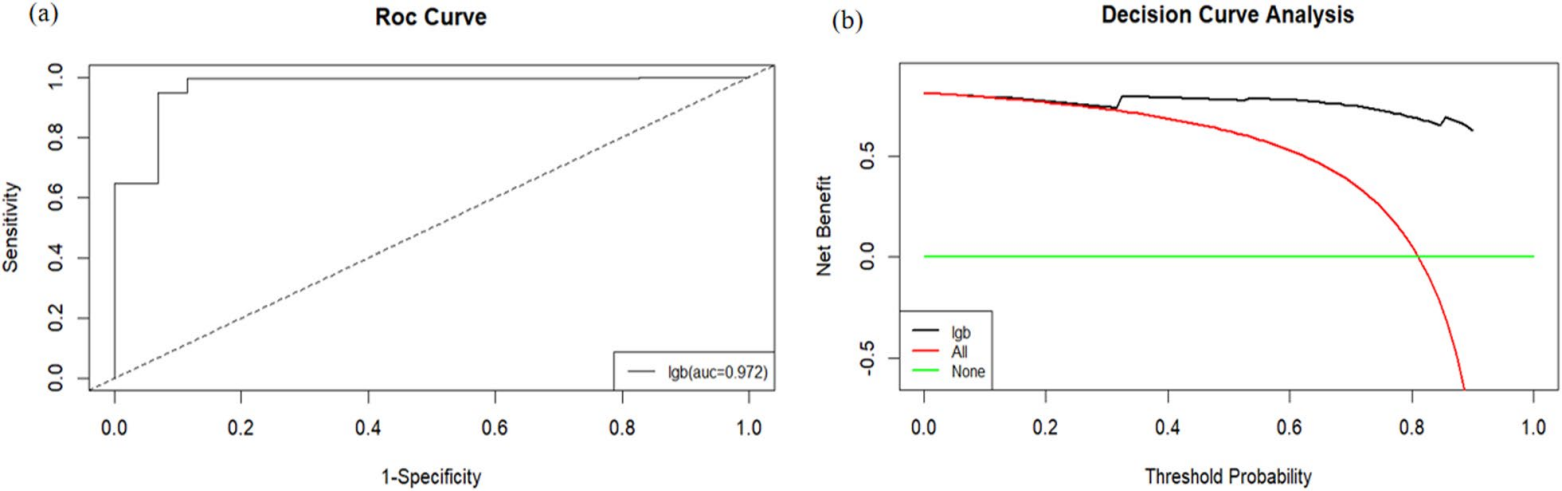
External validation ROC curve for the LGB model **a** showing the model’s performance in distinguishing positive cases; decision curve analysis (DCA) **b** based on the LGB model for external validation. The external validation DCA results based on the LGB model demonstrate that this model exhibits significant net benefits across various threshold probabilities at different time points

After SMOTE analysis, the AUC value of the final model in the external validation set reached 0.972. This is highly consistent with the results of the internal validation results, indicating that the model demonstrates excellent performance in both internal and external validation. The DCA results of the external validation based on the LGB model show that the model has significant net benefits at various threshold probabilities and at different time points, as do the internal validation results (Fig. 5). Fig. S2 illustrates the importance of external features on the basis of the SHAP analysis results.

## Discussion

The aim of this respective, multicenter study was to investigate and compare 10 ML models designed to predict successful weaning from mechanical ventilation. Machine learning techniques have shown great potential in the medical field. Compared to previous studies, this research has constructed 10 machine learning models and compared the advantages and disadvantages of each model, thereby better overcoming the limitations of each model [20]. Ultimately, we identified a set of predictive risk factors and built the optimal predictive model. To assess the performance of the models, we utilized two metrics, IDI and NRI, which are typically used to compare the predictive capabilities of different models to assist researchers in selecting the best predictive model. The higher the values of NRI and IDI, the greater the improvement of the new model over the baseline model, indicating stronger predictive capabilities [21]. The results show that the LGB model significantly outperformed other models in classification accuracy, while also being more advantageous in terms of economy, user-friendliness, and adaptability to different clinical scenarios. Additionally, DCA confirmed that this machine learning model has good clinical applicability and acceptance in the weaning context. Finally, SHAP analysis revealed the most influential predictive factors for determining successful weaning of mechanically ventilated patients, enhancing the interpretability and transparency of current machine learning models. Therefore, this study provides valuable insights for personalized and precise weaning predictions for patients receiving invasive mechanical ventilation support.

On the basis of univariate screening, we used LASSO regression for feature selection modeling. This approach effectively addresses issues of multicollinearity and overfitting among variables. Key factors that predict the withdrawal of invasive mechanical ventilation support were identified [22]. The use of the LASSO method enhances both the predictive capability and the stability of the model. Studies have shown that consciousness, Glasgow Coma Scale (GCS) score, duration of mechanical ventilation, cough strength, age, Acute Physiology and Chronic Health Evaluation II(APACHEII) score, psychological factors, hemoglobin level, Sequential Organ Failure Assessment(SOFA) score, serum albumin level, and certain respiratory parameters are significant predictors of successful weaning [23–26], consistent with the results of our LASSO analysis. However, the insensitivity and specificity of these indicators have also been noted, and studies have reported that these indicators do not consider the influence of other factors, making it imperative to improve the robustness of clinical decision-making [4, 27]. Jia et al. [9] identified several factors that influence weaning outcomes, including the fraction of inspired Oxygen(FiO2), ventilator mode, peak pressure, positive end-expiratory pressure(PEEP), tidal volume, and mean airway pressure. Otaguro [28] reported that duration of mechanical ventilation, FiO2, PEEP, maximum and mean airway pressures, age, and albumin concentration were important predictors of successful weaning. Lin [29] also reported that peak pressure, respiratory rate, and APACHE II score are significant influencing factors. However, these studies did not comprehensively consider all potential clinical influencing factors. This current study incorporates multiple dimensions of factors affecting weaning, including patient demographic characteristics, comorbidities, vital signs, treatment measures, and laboratory indicators obtained from blood gas and biochemical analyses. This comprehensive approach takes into account the multifaceted influences on weaning, thereby avoiding the biases that may have arisen from a narrow focus on weaning factors in previous research.

Current guidelines [24, 30] recommend the use of the spontaneous breathing trial (SBT) as a tool for predicting weaning outcomes. Nevertheless, approximately 10% to 20% of patients who successfully complete an SBT may require reintubation within 48 h after extubation [25]. Various technical methods, including risk prediction nomograms [31], machine learning [20, 32], and a combination of association rule learning and weighted network analysis [33], have been used to analyze and process datasets related to factors or characteristics influencing weaning, leading to the establishment of predictive models. However, researchers often construct risk prediction models on the basis of a single influencing factor, resulting in heterogeneity and a lack of robustness in clinical decision-making. Most models also lack external validation. Furthermore, a few studies have provided explanations of the constructed models.

In the ICU, parameters such as vital signs, biochemical indicators, and ventilator-related metrics are readily available. However, integrating these indicators in a way that properly guides weaning remains a challenge in current research. Compared with traditional predictive models, machine learning offers better predictive performance [34]. Prediction of the success of withdrawal from mechanical ventilation involves both continuous and categorical variables, and this often results in the use of imbalanced and sparse datasets. Our research indicates that the LGB model is optimal for this task. One key advantage of LGB is its ability to handle mixed-type features, this helps preserve the true distribution of these variables within the model. Additionally, LGB uses a histogram-based decision tree approach, discretizing continuous features during the data-splitting process, and thereby allowing for effective management of sparse data. Furthermore, this model is particularly well equipped to address the challenges posed by imbalanced datasets. Although we took steps to mitigate the imbalance during data preprocessing, employing a model that is inherently proficient in managing such issues is more suitable for our objectives [35–37].

To improve the interpretability and intuitiveness of ML models, we applied SHAP values to analyze LGB model. SHAP values provide a consistent and locally accurate assessment of the contribution of each feature to the model’s predictions, enabling a more transparent understanding of the relationships between input variables and outcomes [37, 38]. The detailed insights and risk factor explanations derived from the SHAP analysis offer clinicians deeper insights into the decision-making process, allowing them to make more informed clinical judgments rather than relying solely on algorithmic outputs. The results identified several key factors associated with successful weaning from invasive mechanical ventilation, including serum creatinine levels, lactate levels, consciousness state, SpO₂, systolic blood pressure, cough reflex, chronic respiratory disease diastolic blood pressure, and age. In clinical practice, these findings highlight the importance of considering both positive and negative influencing factors revealed by SHAP analysis when making extubation decisions, thereby facilitating more personalized and evidence-based treatment strategies.

## Limitations

Overall, the machine learning-driven prediction model for the successful weaning of patients from mechanical ventilation in the ICU offers an efficient and cost-effective approach to the prediction of successful extubation. However, this study has several limitations, which we have identified as follows. First, in recent years, against the backdrop of precision medicine and the deepening of critical care ultrasound research, ultrasound has emerged as a noninvasive method of measuring diaphragmatic displacement (DD) and diaphragmatic thickness (DT), thus garnering widespread attention [39, 40]. Numerous studies [41, 42] have shown that both DD and diaphragmatic thickness fraction (DTF) can accurately reflect diaphragmatic function. However, this study did not include imaging data among the possible factors influencing weaning. Second, in critically ill patients, the assessment of volume status involves multiple complex scenarios. Clinically, to accurately evaluate a patient’s hemodynamic status, it is essential to integrate multidimensional indicators in conjunction with the patient’s underlying condition. Invasive monitoring methods are considered the “gold standard” for assessing a patient’s volume status and are particularly suitable in cases involving severely ill patients. These methods include measurement of central venous pressure (CVP) and pulmonary capillary wedge pressure (PCWP), and pulse indicator continuous cardiac output (PiCCO) monitoring [43]. However, during the collection of data for this study, obtaining these invasive indicators posed significant challenges. Therefore, more readily available clinical parameters, such as urine output, NT-proBNP, BNP, serum potassium, sodium, blood urea nitrogen, and creatinine, were used to construct a predictive model in this study. Third, the assessment of respiratory muscle strength plays a crucial role in guiding weaning from mechanical ventilation [44]. This assessment typically includes measurement of parameters such as maximal inspiratory pressure (MIP), maximal expiratory pressure (MEP), and cough peak flow (CPF). However, in intubated patients, evaluation of respiratory muscle strength can be challenging because of factors such as the patient’s level of consciousness, limitations in airway management, insufficient cooperation, and the complexity of operating the required equipment. These challenges may result in incomplete data collection or compromise the reliability of the results. In this study, the cough ability index was used as a basis for assessing ventilatory muscle strength. Fourth, the model described here has not yet been widely adopted in multicenter clinical practice; this represents an important direction for future research.

## Conclusions

The optimized predictive model, developed through the integration of multidimensional predictive factors and diverse machine learning algorithms, exhibits superior predictive accuracy and demonstrates significant clinical potential for determining the optimal weaning timing in patients receiving invasive mechanical ventilation.

## Supplementary Information

Supplementary Material 1.

## Data Availability

The datasets generated and/or analyzed during the current study are not publicly available but are available from the corresponding author upon reasonable request. (xiaopingzhu0424@163.com).

## Abbreviations

ICU: Intensive care unit
ML: Machine learning
AI: Artificial intelligence
SHAP: SHapley Additive exPlanations
DCA: Decision curve analysis
NRI: Net reclassification improvement
IDI: Integrated discrimination improvement
ECMO: Extracorporeal membrane oxygenation
NIV: Noninvasive ventilation
MICE: Multiple imputation by chained equations
KNN: K-nearest neighbors
SVM: Support vector machine
NN: Neural network
XGB: Extreme gradient boosting
LR: Logistic regression
RF: Random forest
DT: Decision tree
ET: Extra trees
SGD: Stochastic gradient descent
LGB: Light gradient boosting
TP: True positive
TN: True negative
FP: False positive
FN: False negative
LASSO: Least absolute shrinkage and selection operator
pH: Potential of hydrogen
PO₂: Partial pressure of oxygen
Ca: Calcium
K: Potassium
cTnT: Cardiac troponin T
NT-proBNP: N-terminal Pro B-type natriuretic peptide
PCT: Procalcitonin
GCS: Glasgow Coma Scale
APACHE II: Acute Physiology and Chronic Health Evaluation II
SOFA: Sequential Organ Failure Assessment
FiO₂: Fraction of inspired oxygen
PEEP: Positive end-expiratory pressure
SBT: Spontaneous breathing trial
DD: Diaphragmatic displacement
DT: Diaphragmatic thickness
DTF: Diaphragmatic thickness fraction
CVP: Central venous pressure
PCWP: Pulmonary capillary wedge pressure
PiCCO: Pulse indicator continuous cardiac output
MIP: Maximal inspiratory pressure
MEP: Maximal expiratory pressure
CPF: Cough peak flow
