# Supplementary material for: Machine learning-driven prediction model for successful weaning of patients from mechanical ventilation in ICU
Source: Intensive Care Med Exp. 2026 Jan 21;14:6. doi: 10.1186/s40635-026-00859-8 (PMC12824072; doi:10.1186/s40635-026-00859-8)
Supplement: Supplementary file 1 — Supplementary Material 1. [file 40635_2026_859_MOESM1_ESM.docx]

Legend：

Fig.S1: Distribution of LASSO coefficients for 34 predictive factors (b) with parameter λ adjusted using 10-fold cross-validation

Fig.S2: (External Validation)SHAP summary bar plot，Feature importance ranking(a);Feature importance based on SHAP results. The vertical axis represents the features, while the horizontal axis represents the SHAP values. The colors of the points for each feature value are completely different; pink indicates a positive correlation with successful withdrawal, while blue indicates a negative correlation with successful withdrawal(b)

Table S1: Demographic and clinical characteristics of the training and the internal validation groups

Table S2: Demographic and clinical characteristics between derivation and enternal validation groups

Table S3: Important predictive factors for mechanical ventilation patients after LASSO regression analysis

**
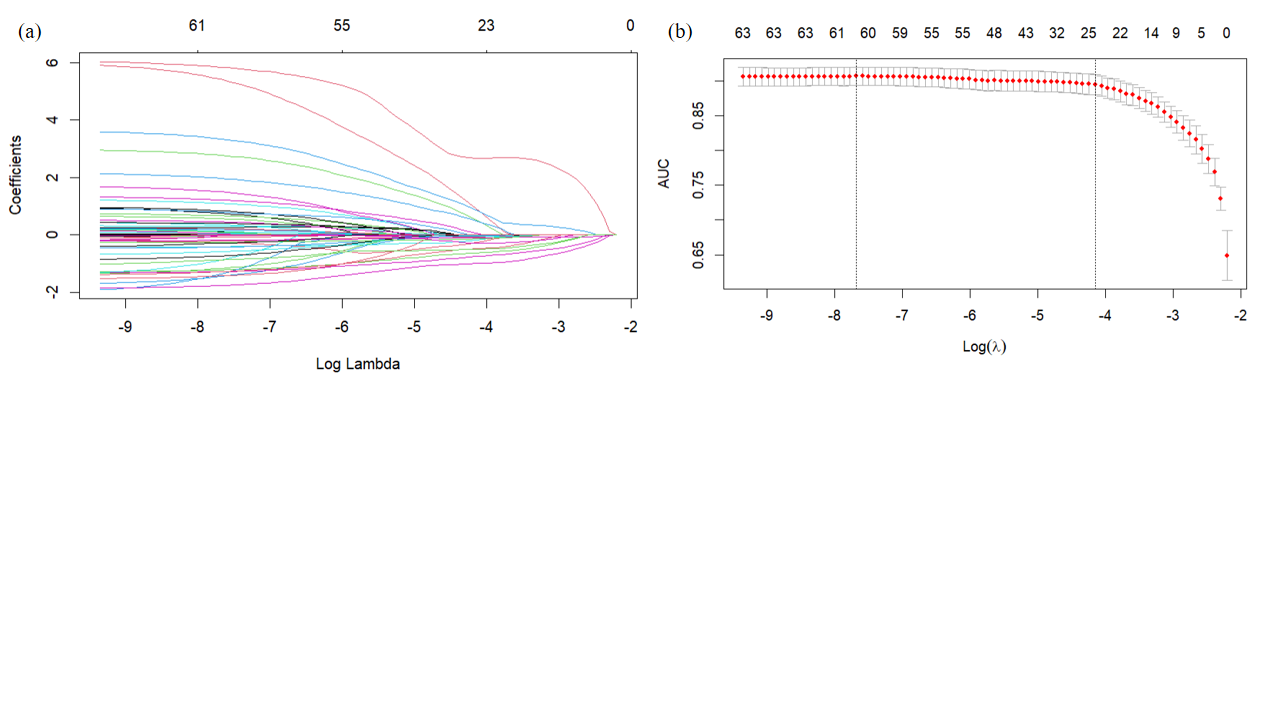
**

**Fig.S1：**Distribution of LASSO coefficients for 34 predictive factors (b) with parameter λ adjusted using 10-fold cross-validation


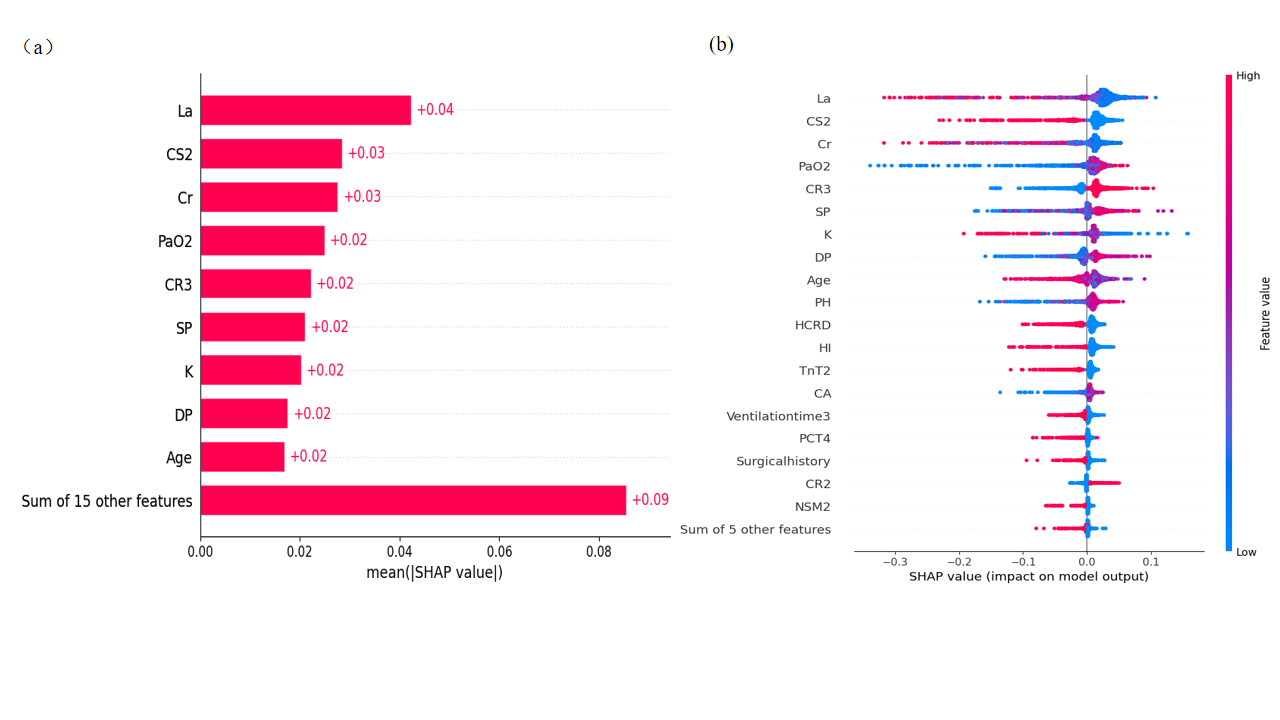


**Fig.S2**: (External Validation)SHAP summary bar plot，Feature importance ranking(a)；Feature importance based on SHAP results. The vertical axis represents the features, while the horizontal axis represents the SHAP values. The colors of the points for each feature value are completely different; pink indicates a positive correlation with successful withdrawal, while blue indicates a negative correlation with successful withdrawal(b).

| **Table S1** Demographic and clinical characteristics of the training and the internal validation groups | | | |
| --- | --- | --- | --- |
| **Variables** | **Training** | **Verification** | ***P*** |
| N | 960 | 411 |  |
| General |  |  |  |
| Age | 66.30±14.20 | 66.40±14.14 | 0.909 |
| Gender |  |  | 0.798 |
| Male, n(%) | 659 (68.65%) | 285 (69.34%) |  |
| Female, n(%) | 301 (31.35%) | 126 (30.66%) |  |
| BMI, n (%) | | | |
| < 18.5 | 125 (13%) | 65 (15.8%) | 0.569 |
| 18.5 ≤ BMI < 24.9 | 606 (63.1%) | 245 (59.6%) |  |
| 25 ≤ BMI < 29.9 | 163 (17%) | 75 (18.2%) |  |
| ≥30 | 66 (6.9%) | 26 (6.3%) |  |
| Marriage status, n (%) | | | |
| Married | 853 (88.9%) | 367 (89.3%) | 0.812 |
| Other | 107 (11.1%) | 44 (10.7%) |  |
| ICU time, n (%) | | | |
| 2–7 d | 382 (39.8%) | 159 (38.7%) | 0.733 |
| 7–28 d | 508 (52.9%) | 222 (54%) |  |
| >28 d | 70 (7.3%) | 30 (7.3%) |  |
| Ventilation time, n (%) | | | |
| 1–3 d | 356 (37.1%) | 147 (35.8%) | 0.836 |
| 3–7 d | 196 (20.4%) | 90 (21.9%) |  |
| ≥7 d | 408 (42.5%) | 174 (42.3%) |  |
| Surgical history, n (%) | | | |
| No | 690 (71.9%) | 270 (65.7%) | 0.052 |
| Yes | 270 (28.1%) | 141 (34.3%) |  |
| Alcohol consumption history , n (%) | | | |
| No | 906 (94.4%) | 383 (93.2%) | 0.396 |
| Yes | 54 (5.6%) | 28 (6.8%) |  |
| Smoking history , n (%) | | | |
| No | 873 (90.9%) | 369 (89.8%) | 0.502 |
| Yes | 87 (9.1%) | 42 (10.2%) |  |
| Heart disease, n (%) | | | |
| No | 555 (57.8%) | 248 (60.3%) | 0.384 |
| Yes | 405 (42.2%) | 163 (39.7%) |  |
| HCRD , n (%) | | | |
| No | 649 (67.6%) | 285 (69.3%) | 0.527 |
| Yes | 311 (32.4%) | 126 (30.7%) |  |
| Diabetes, n (%) | | | |
| No | 740 (77.1%) | 319 (77.6%) | 0.830 |
| Yes | 220 (22.9%) | 92 (22.4%) |  |
| Hypertension, n (%) | | | |
| No | 538 (56%) | 235 (57.2%) | 0.698 |
| Yes | 422 (44%) | 176 (42.8%) |  |
| Hemodynamic instability , n (%) | | | |
| No | 816 (85%) | 348 (84.7%) | 0.876 |
| Yes | 144 (15%) | 63 (15.3%) |  |
| Pulmonary arterial hypertension, n (%) | | | |
| No | 729 (75.9%) | 317 (77.1%) | 0.635 |
| Yes | 231 (24.1%) | 94 (22.9%) |  |
| Comorbid neurological disorders, n (%) | | | |
| No | 721 (75.1%) | 306 (74.5%) | 0.799 |
| Yes | 239 (24.9%) | 105 (25.5%) |  |
| kidney diseases, n (%) | | | |
| No | 874 (91%) | 374 (91%) | 0.979 |
| Yes | 86 (9%) | 37 (9%) |  |
| Vital Signs | | | |
| Muscle strength (grade), n (%) | | | |
| 0 | 15 (1.6%) | 2 (0.5%) | 0.727 |
| I & II&III & IV | 100 (10.4%) | 45 (10.9%) |  |
| V | 845 (88%) | 364 (88.6%) |  |
| State of consciousness , n (%) | | | |
| Alert | 638 (66.5%) | 277 (67.4%) | 0.657 |
| Drowsy & Stupor & Coma | 231 (24.1%) | 100 (24.3%) |  |
| Agitated&Sedated | 91 (9.5%) | 34 (8.3%) |  |
| heart rate,beats,per minute | 89.44±17.63 | 89.28±16.69 | 0.876 |
| Systolic pressure, mmHg | 128.93±20.78 | 127.22±19.73 | 0.156 |
| Diastolic pressure, mmHg | 68.46±11.55 | 68.34±11.51 | 0.859 |
| Body temperature, ℃ | 36.88±0.59 | 36.84±0.54 | 0.196 |
| RSBI | 38.51±10.31 | 38.52±8.89 | 0.992 |
| Blood oxygen saturation | 98.25±7.35 | 97.84±5.10 | 0.304 |
| Cough reflex, n (%) | | | |
| 0 | 101 (10.5%) | 48 (11.7%) | 0.958 |
| 1 | 38 (4%) | 23 (5.6%) |  |
| 2 | 232 (24.2%) | 84 (20.4%) |  |
| 3 | 589 (61.4%) | 256 (62.3%) |  |
| Sputum viscosity, n (%) | | | |
| I | 502 (52.3%) | 226 (55%) | 0.393 |
| II | 424 (44.2%) | 170 (41.4%) |  |
| III | 34 (3.5%) | 15 (3.6%) |  |
| Therapeutic Measures | | | |
| Early rehabilitation (during the intubation period), n (%) | | | |
| No | 912 (95%) | 391 (95.1%) | 0.917 |
| Yes | 48 (5%) | 20 (4.95%) |  |
| Systemic steroid treatmen, n (%) | | | |
| No | 949 (98.9%) | 405 (98.5%) | 0.630 |
| Yes | 11 (1.1%) | 6 (1.5%) |  |
| Use of Positive Inotropic and Vasoactive Drugs, n (%) | | | |
| No | 609 (63.4%) | 256 (62.3%) | 0.686 |
| Yes | 351 (36.6%) | 155 (37.7%) |  |
| Use of Sedatives, n (%) | | | |
| No | 553 (57.6%) | 242 (58.9%) | 0.661 |
| Yes | 407 (42.4%) | 169 (41.1%) |  |
| Nutritional support method, n (%) | | | |
| Enteral | 573 (59.7%) | 246 (59.9%) | 0.996 |
| Enteral and Parenteral | 224 (23.3%) | 93 (22.6%) |  |
| Parenteral | 100 (10.4%) | 43 (10.5%) |  |
| Nothing by mouth | 63 (6.6%) | 29 (7.1%) |  |
| Laboratory Indicators | | | |
| pH | 7.41±0.08 | 7.41±0.11 | 0.887 |
| PaO_2_, mmHg | 130.94±47.04 | 131.13±47.69 | 0.946 |
| Calcium, mmol/L | 1.17±0.10 | 1.17±0.08 | 0.924 |
| PaCO₂, mmHg | 41.24±7.55 | 41.36±8.54 | 0.803 |
| Lactate, mmHg | 2.27±2.31 | 2.49±2.62 | 0.116 |
| Glucose, mmol/L | 10.08±4.38 | 9.95±4.20 | 0.616 |
| Hemoglobin,g/dL | 91.52±25.67 | 90.58±26.83 | 0.537 |
| Potassium, mmol/L | 4.05±1.04 | 3.96±0.80 | 0.142 |
| Sodium(Na), mmol/L | 141.45±6.84 | 141.77±7.51 | 0.441 |
| White blood cells, *10^9/L | 15.29±67.89 | 13.49±52.12 | 0.631 |
| Albumin, g/L | 33.51±5.88 | 33.27±6.03 | 0.491 |
| Creatinine, μmol/L | 108.63±161.74 | 106.20±103.82 | 0.779 |
| Troponin T, μg/L, n% | | | |
| <0.1 | 711 (74.1%) | 305 (74.2%) | 0.896 |
| 0.1–1.0 | 199 (20.7%) | 77 (18.7%) |  |
| >1.0 | 50 (5.2%) | 29 (7.1%) |  |
| NT-proBN, ng/L, n% | | | |
| <125 | 255 (26.6%) | 113 (27.5%) | 0.336 |
| 125–450 | 176 (18.3%) | 59 (14.4%) |  |
| 450–900 | 125 (13%) | 47 (11.4%) |  |
| >900 | 404 (42.1%) | 192 (46.7%) |  |
| Procalcitonin, ng/L, n% | | | |
| <0.1 | 122 (12.7%) | 51 (12.4%) | 0.079 |
| 0.1–0.5 | 505 (52.6%) | 193(47.0%) |  |
| 0.5–2 | 169 (17.6%) | 83 (20.2%) |  |
| >2 | 164 (17.1%) | 84 (20.4%) |  |
| Data are presented as mean (SD), median [IQR], or n (%) | | | |

| **Table S2** Demographic and clinical characteristics between derivation and enternal validation groups | | | |
| --- | --- | --- | --- |
| **Variables** | **Derivation** | **External validation** | ***P*** |
| N | 1371 | 402 |  |
| General | | | |
| Age | 66.331±4.17 | 65.84±15.18 | 0.546 |
| Gender |  |  | 0.584 |
| Male,n (%) | 944 (68.9%) | 271 (67.4%) |  |
| Female,n (%) | 427 (31.1%) | 131 (32.6%) |  |
| BMI,n (%) | | | |
| < 18.5 | 190 (13.9%) | 53 (13.2%) | *0.008 |
| 18.5 ≤ BMI < 24.9 | 851 (62.1%) | 225 (56%) |  |
| 25 ≤ BMI < 29.9 | 238 (17.4%) | 81 (20.1%) |  |
| ≥30 | 92 (6.7%) | 43 (10.7%) |  |
| Marriage status, n (%) | | | |
| Married | 1220 (89%) | 377 (93.8%) | *0.005 |
| Other | 151 (11%) | 25 (6.2%) |  |
| ICU time, n (%) | | | |
| 2–7 d | 541 (39.5%) | 186 (46.3%) | *0.011 |
| 7–28 d | 730 (53.2%) | 194 (48.3%) |  |
| >28 d | 100 (7.3%) | 22 (5.5%) |  |
| Ventilation time, n (%) | | | |
| 1–3 d | 503 (36.7%) | 151 (37.6%) | 0.161 |
| 3–7 d | 286 (20.9%) | 105 (26.1%) |  |
| ≥7 d | 582 (42.5%) | 146 (36.3%) |  |
| Surgical history, n (%) | | | |
| No | 960 (70%) | 356 (88.6%) | <0.001 |
| Yes | 411 (30%) | 46 (11.4%) |  |
| Alcohol consumption history , n (%) | | | |
| No | 1289 (94%) | 371 (92.3%) | 0.212 |
| Yes | 82 (6%) | 31 (7.7%) |  |
| Smoking history , n (%) | | | |
| No | 1242 (90.6%) | 376 (93.5%) | 0.066 |
| Yes | 129 (9.4%) | 26 (6.5%) |  |
| Heart disease, n (%) | | | |
| No | 803 (58.6%) | 248 (61.7%) | 0.263 |
| Yes | 568 (41.4%) | 154 (38.3%) |  |
| HCRD , n (%) | | | |
| No | 934 (68.1%) | 283 (70.4%) | 0.388 |
| Yes | 437 (31.9%) | 119 (29.6%) |  |
| Diabetes, n (%) | | | |
| No | 1059 (77.2%) | 393 (97.8%) | <0.001 |
| Yes | 312 (22.8%) | 9 (2.2%) |  |
| Hypertension, n (%) | | | |
| No | 773 (56.4%) | 353 (87.8%) | <0.001 |
| Yes | 598 (43.6%) | 49 (12.2%) |  |
| Hemodynamic instability ,n(%) | | | |
| No | 1164 (84.9%) | 351 (87.3%) | 0.228 |
| Yes | 207 (15.1%) | 51 (12.7%) |  |
| Pulmonary arterial hypertension, n (%) | | | |
| No | 1046 (76.3%) | 263 (65.4%) | <0.001 |
| Yes | 325 (23.7%) | 139 (34.6%) |  |
| Comorbid neurological disorders, n (%) | | | |
| No | 1027 (74.9%) | 270 (67.2%) | *0.002 |
| Yes | 344 (25.1%) | 132 (32.8%) |  |
| kidney diseases, n (%) | | | |
| No | 1248 (91%) | 394 (98%) | <0.001 |
| Yes | 123 (9%) | 8 (2%) |  |
| Vital Signs | | | |
| Muscle strength (grade), n (%) | | | |
| 0 | 17 (1.2%) | 29 (7.2%) | 0.486 |
| I & II&III & IV | 145 (10.6%) | 1 (0.2%) |  |
| V | 1209 (88.2%) | 372 (92.5%) |  |
| State of consciousness, n (%) | | | |
| Alert | 915 (66.7%) | 241 (60%) | *0.007 |
| Drowsy & Stupor & Coma | 331 (24.1%) | 111 (27.6%) |  |
| Agitated&Sedated | 125 (9.1%) | 50 (12.4%) |  |
| Heart rate, beats, per minute | 89.39±17.35 | 88.01±16.96 | 0.079 |
| Systolic pressure, mmHg | 128.42±20.48 | 130.03±18.49 | 0.077 |
| Diastolic pressure, mmHg | 68.43±11.53 | 68.32±12.69 | 0.437 |
| Body temperature, ℃ | 36.87±0.57 | 36.87±0.78 | 0.476 |
| RSBI | 38.52±9.90 | 38.38±12.86 | 0.819 |
| Blood oxygen saturation | 98.12±6.76 | 98.88±2.20 | *0.027 |
| cough reflex, n (%) | | | |
| 0 | 149 (10.9%） | 65 (16.2%) | 0.254 |
| 1 | 61 (4.4%） | 9 (2.2%） |  |
| 2 | 316 (23%） | 20 (5%） |  |
| 3 | 845 (61.6%） | 308 (76.6%） |  |
| Sputum viscosity, n (%) | | | |
| I | 728 (53.1%） | 223 (55.5%） | 0.815 |
| II | 594 (43.3%） | 152 (37.8%） |  |
| III | 49 (3.6%） | 27 (6.7%） |  |
| Therapeutic Measures | | | |
| Early rehabilitation(during the intubation period), n (%) | | | |
| No | 1303 (95%） | 373 (92.8%） | 0.081 |
| Yes | 68 (5%） | 29 (7.2%） |  |
| Systemic steroid treatmen, n (%) | | | |
| No | 1354 (98.8%） | 400 (99.5%） | 0.204 |
| Yes | 17 (1.2%） | 2 (0.5%） |  |
| Use of Positive Inotropic and Vasoactive Drugs, n (%) | | | |
| No | 865 (63.1%） | 251 (62.4%） | 0.811 |
| Yes | 506 (36.9%） | 151 (37.6%） |  |
| Use of Sedatives, n (%) | | | |
| No | 795 (58%） | 335 (83.3%） | <0.001 |
| Yes | 576 (42%） | 67 (16.7%） |  |
| Nutritional support method, n (%) | | | |
| Enteral | 819 (59.7%） | 253 (62.9%） | 0.537 |
| Enteral and Parenteral | 317 (23.1%） | 71 (17.7%） |  |
| Parenteral | 143 (10.4%） | 34 (8.5%） |  |
| Nothing by Mouth | 92 (6.7%） | 44 (10.9%） |  |
| Laboratory Indicators | | | |
| PH | 7.41±0.09 | 7.42±0.07 | *0.041 |
| PaO_2_, mmHg | 131±47.22 | 135.06±44.42 | 0.125 |
| Calcium, mmol/L | 1.17±0.10 | 1.18±0.07 | <0.001 |
| PaCO₂, mmHg | 41.27±7.86 | 40.37±6.57 | *0.035 |
| Lactate, mmHg | 2.34±2.41 | 1.79±1.40 | <0.001 |
| Glucose, mmol/L | 10.04±4.33 | 9.17±3.66 | <0.001 |
| Hemoglobin, g/dL | 91.24±26.02 | 93.98±21.91 | 0.054 |
| Potassium, mmol/L | 4.02±0.98 | 4.09±1.41 | 0.260 |
| Sodium(Na), mmol/L | 141.54±7.05 | 141.48±6.35 | 0.869 |
| White Blood Cell, *10^9/L | 14.75±63.56 | 10.29±5.20 | 0.160 |
| Albumin, g/L | 33.43±5.93 | 33.87±5.22 | 0.183 |
| Creatinine, μmol/L | 107.90±146.76 | 88.49±61.99 | <0.001 |
| Troponin T, μg/L, n % | | | |
| <0.1 | 1016(74.1%) | 343(85.3%) | <0.001 |
| 0.1–1.0 | 276(20.1%) | 53(13.2%) |  |
| >1.0 | 79(5.8%) | 6(1.5%) |  |
| NT-proBN, ng/L, n % | | | |
| <125 | 368(26.8%) | 157(39.1%) | <0.001 |
| 125–450 | 235(17.1%) | 59(14.7%) |  |
| 450–900 | 172(12.5%) | 56(13.9%) |  |
| >900 | 596(43.5%) | 130(32.3%) |  |
| Procalcitonin, ng/L, n % | | | |
| <0.1 | 173(12.6%) | 193(48%) | <0.001 |
| 0.1–0.5 | 698(50.9%) | 91(22.6%) |  |
| 0.5–2 | 252(18.4%) | 57(14.2%) |  |
| >2 | 248(18.1%) | 61(15.2%) |  |
| Data are presented as mean (SD), median [IQR], or n (%)；*Statistically significant (P < 0.05) | | | |

**Table S3** Important predictive factors for mechanical ventilation patients after LASSO regression analysis

| **Variables** |  |  |  |
| --- | --- | --- | --- |
| Age | HCRD | Nutritional support method | K |
| Marriage | Hemodynamic instability | PH | Cr |
| ICU time | Consciousness state | PaO2 | TnT |
| Ventilation time | Systolic pressure | Calcium | NT-proBN |
| Surgical history | Diastolic pressure | Lactate | PCT |
| Heart disease | Cough reflex |  |  |
| *Important predictive factors after Lasso regression analysis | | | |
